# S-Doped NiFe_2_O_4_ Nanosheets Regulated Microbial Community of Suspension for Constructing High Electroactive Consortia

**DOI:** 10.3390/nano12091496

**Published:** 2022-04-28

**Authors:** Jiaxin Li, Bo Song, Chongchao Yao, Zhihao Zhang, Lei Wang, Jing Zhang

**Affiliations:** 1 National Engineering Laboratory for VOCs Pollution Control Material and Technology, Research Center for Environmental Material and Pollution Control Technology, University of Chinese Academy of Sciences, Beijing 101408, China; lijiaxin194@mails.ucas.ac.cn (J.L.); ancico@126.com (C.Y.); zhzhang_st@rcees.ac.cn (Z.Z.); 2 Sino-Danish College, University of Chinese Academy of Sciences, Beijing 100049, China; 3 Key Laboratory of Environmental Biotechnology, Research Center for Eco-Environmental Sciences, Chinese Academy of Sciences, Beijing 100085, China; bosong_st@rcees.ac.cn; 4 College of Resources and Environment, University of Chinese Academy of Sciences, Beijing 100049, China

**Keywords:** microbial fuel cells, S-doping anode, planktonic bacteria, biofilms, interactions

## Abstract

Iron-based nanomaterials (NMs) are increasingly used to promote extracellular electron transfer (EET) for energy production in bioelectrochemical systems (BESs). However, the composition and roles of planktonic bacteria in the solution regulated by iron-based NMs have rarely been taken into account. Herein, the changes of the microbial community in the solution by S-doped NiFe_2_O_4_ anodes have been demonstrated and used for constructing electroactive consortia on normal carbon cloth anodes, which could achieve the same level of electricity generation as NMs-mediated biofilm, as indicated by the significantly high voltage response (0.64 V) and power density (3.5 W m^−2^), whereas with different microbial diversity and connections. Network analysis showed that the introduction of iron-based NMs made *Geobacter* positively interact with *f_Rhodocyclaceae*, improving the competitiveness of the consortium (*Geobacter* and *f_Rhodocyclaceae*). Additionally, planktonic bacteria regulated by S-doped anode alone cannot hinder the stimulation of *Geobacter* by electricity and acetate, while the assistance of lining biofilm enhanced the cooperation of sulfur-oxidizing bacteria (SOB) and fermentative bacteria (FB), thus promoting the electroactivity of microbial consortia. This study reveals the effect of S-doped NiFe_2_O_4_ NMs on the network of microbial communities in MFCs and highlights the importance of globality of microbial community, which provides a feasible solution for the safer and more economical environmental applications of NMs.

## 1. Introduction

Extracellular electron transfer (EET) enables some microorganisms to transfer electrons to extracellular minerals or organic matter, generating energy to maintain their growth [1,2]. EET has been widely employed to construct microbial fuel cells (MFCs) and other bioelectrochemical systems (BESs), which shows an important application prospect in wastewater treatment, electricity generation, bioremediation, and bioproduction [3,4,5,6]. In MFCs, the electrochemically active bacteria (EAB) oxidize reduced substrates and release electrons via EET; then, the electrons flow to the cathode through an external circuit [7]. Anode as the habitat for microorganisms is a critical factor in achieving high-performance MFCs. EET process is influenced by the electron transfer at the bacteria–electrode interface or biointerface. The chemical structure and surface functionality of the anode materials directly affect EET efficiency. Carbon-based electrodes have been widely used as MFC anodes due to their advantages, such as low cost, good electrical conductivity, good biocompatibility, and strong corrosion resistance [8,9]. However, due to its hydrophobicity, it is not conducive to microbial adhesion, which often leads to poor electron transfer capacity, thus causing low power output.

In situ grown nanostructures to surface modification of carbon-based materials are mainly used for facilitating EET, further enhancing MFCs performance [10,11,12,13]. Iron-based nanomaterials (NMs) play multiple functions in promoting EET [14,15,16] due to EAB can discharge electrons by reducing insoluble Fe (III) [17], and the introduction of Fe ions always promotes the enrichment of *Geobacter*, one of the most extensively studied EAB to date [18,19]. Moreover, Iron oxides (Fe_x_O_y_) can act as electron conduits [20,21], conductive assistants [22], or electron reservoirs [14] to facilitate interspecies electron transfer (IET), which simultaneously improves the potential of bioremediation. Recent studies have shown that NiFe_2_O_4_ exhibits superior electrocatalytic activity and conductivity compared to Fe_x_O_y_ because of the presence of variable valent cations [23]. Additionally, the electrocatalyst (S-NiFe_2_O_4_) generated through a vacancy filling strategy exhibits higher catalytic activity and durability in both alkaline and neutral pH than the NiFe_2_O_4_ electrocatalyst [24,25], indicating S-NiFe_2_O_4_ electrode material has great potential as an MFC anode for electricity generation.

Diverse microbial consortia exist in natural environments, and many artificial systems, the interactions between the microbial partners in these mixed cultures have a significant impact on the combined performance of microbial consortia [26]. For example, the aerobic *Bacillus* positively interacts with the anaerobic *Clostridium*, significantly enhancing hydrogen production [27]. The “interspecies ecological communication” of *Pseudomonas aeruginosa* and *Klebsiella variicola* can enhance the electrochemical activity in MFCs [28]. Microbes in MFCs have spatial distribution heterogeneity in the whole chamber. Some studies have revealed microbial communities away from the electrodes also contribute significantly to EET and energy production [29,30]. In particular, the existence of abundant planktonic bacteria in the bulk solution (the suspension community) is also responsible for the essential source of energy production. Therefore, constructing microbial consortia based on microbial interaction regulated by NMs could be one of the possible breakthroughs for the further environmental application of MFCs. Nevertheless, the effects of S-NiFe_2_O_4_ on the diversity and network relationships of microbial communities in the whole chamber are still unclear.

This study focused on the effect of S-doped NiFe_2_O_4_ nanosheet arrays on carbon cloth (S-NFO@CC) as MFC anodes on microbial community in the solution and used it to construct efficient electroactive consortia. To achieve this goal, we compared the electricity generation performance of MFCs with different S10-NFO@CC nanomaterials, then used the suspension community with the best MFC performance for building up different MFCs, and the outcomes were evaluated in terms of voltage response and power density. Finally, the microbial diversity and interactions of different strategies were analyzed.

## 2. Materials and Methods

### 2.1. Synthesis of the S-NFO@CC Nanosheets

Carbon cloth (CC, WOS1009, 330 µm thickness, CeTech Co., Ltd., Taiwan, China) was used as the substrate after ultrasonicating it in acetone, ethanol, and deionized (DI) water for 30 min each. A mixture of Ni (NO_3_)_2_·6H_2_O (0.0873 g), Fe (NO_3_)_3_·9H_2_O (0.2424 g), NH_4_F (0.0527 g), and Urea (CH_4_N_2_O, 0.0902 g) was added into DI water (40 mL) stirring for 30 min. Subsequently, 15 mL mixed solution and a piece of CC with a size of 1 × 1 cm^2^ were transferred into a 23 mL Teflon-lined stainless steel autoclave, which was sealed and maintained at 120 °C for 6 h. After cooling to room temperature, the nickel-iron layered double hydroxide modified CC (NiFe-LDH@CC) was removed from the solution and washed several times with DI water and ethanol. The precursor was then dried at 60 °C overnight and annealed at 400 °C or 700 °C for 1 h with a heating rate of 5 °C min^−1^ in an N_2_ atmosphere to obtain the NiFe_2_O_4_ nanocrystals (NFO400@CC and NFO700@CC). Then 15 mL sulfide precursor solutions (10 mM Na_2_S·9H_2_O) and a piece of NFO@CC were transferred into the 23 mL Teflon-line stainless steel autoclave and maintained at 120 °C for 6 h or 8 h. The S-doped NiFe_2_O_4_ nanosheet arrays on CC was washed with DI water, dried at 60 °C after the sulfide conversion process, and finally marked as S10-NFO400@CC, S10(T8)-NFO400@CC, and S10-NFO700@CC.

### 2.2. Material Characterization and MFC Characterization

The morphology of materials was characterized by scanning electron microscopy (SEM, JEOL JSM-7900F, Tokyo, Japan) equipped with an acceleration voltage of 15.0 kV. XRD analysis was performed by Bruker D8 advance (Karlsruhe, Germany) with Cu Kα radiation (λ = 0.1542 nm) under a voltage of 40 kV and a current of 40 mA.

Two-chamber MFCs (WENOOTE Inc., Chuzhou, China) with 1 kΩ external resistance operated at 37 °C in repeated batch mode. The anode chamber and cathode chamber with the same working volume (50 mL) were separated by a proton exchange membrane (Nafion 117, Dupont Co., Wilmington, DE, USA). Each anode chamber which was equipped with either CC, NFO400@CC, S10-NFO@CC anode, was inoculated with 5.0 mL of pre-acclimated bacteria deriving originally from activated anaerobic sludge and fed with a medium solution (pH 7.0) containing 50 mM phosphate buffer solution (PBS), sodium acetate (CH_3_COONa, 2 g L^−1^), trace minerals (12.5 mL L^−1^), and vitamins (5 mL L^−1^) as described before [11]. Thereinto, the main ions of PBS solution include Na^+^, K^+^, H^+^, HPO_4_^2−^, PO_4_^3−^ and Cl^−^. Trace minerals mainly contain Mg^2+^, Mn^2+^, Na^+^, SO_4_^2−^, Cl^−^, etc. Vitamins mainly contain organics such as pyridoxine hydrochloride, riboflavin, Vitamin B12, Nicotinic acid, etc. Each cathode chamber was equipped with a commercially available 3D carbon fiber brush electrode and fed with 50 mM KCl and 50 mM K_3_[Fe (CN)_6_] solution. The anode solution was refreshed and purged with N_2_ for 15 min to keep anaerobic conditions when the operating voltage dropped below 0.05 V. Cell voltages of MFCs were recorded every 10 min by a digital multimeter (DAQ2650 with 7701 modules, Keithley Instruments Inc., Cleveland, OH, USA). Electrochemical polarization and power density curves were acquired by varying the external resistance at a range of 2–0.2 kΩ when the MFCs produced stable maximum voltages after four steady and repeatable voltage cycles. Maximum current density and maximum power density were normalized to the electrode surface area (calculated as a double side, 2 cm^2^).

### 2.3. Different Strategies of MFC Operation

After 1 month of operation of MFCs with CC and S10-NFO400@CC anodes, the bacteria in the bulk solution of these two anode chambers were collected, respectively, denoted planktonic_CC and planktonic_S10. As summarized in Table 1, Group 1 as a control and three other strategies were employed to construct conductive microbes using a common CC anode. New MFC with a new CC anode was inoculated with planktonic_CC (denoted Group 2) and planktonic_S10 (denoted Group 3), respectively. In addition, a reactor operated for about four months in Group 1 was replaced with a new CC anode and inoculated with planktonic_S10 as Group 4. All MFCs of four groups were operated and tested using the same electrolytes and methods shown in Section 2.2.

### 2.4. Microbial Community Analysis

In order to analyze the effect of S10-NFO400@CC anode on the microbial community structure and composition, anode biofilm samples and planktonic bacteria were collected at the end of the operation and analyzed by 16S rRNA high-throughput sequencing. The total DNA extraction was performed using a PowerSoil DNA Isolation Kit (MoBio PowerSoil, Carlsbad, CA, USA). DNA concentration was confirmed by a spectrophotometer (NanoDrop 2000c, Thermo Fisher Scientific, Waltham, MA, USA). The universal PCR primers of Forward 338F (5′-AYTGGGYDTAAAGVG-3′) and Reverse 806R (5′-GGACTACHVGGGTWTCTAAT-3′) were used for the amplification of V3-V4 hypervariable regions of 16S rRNA genes by thermocycler PCR system (GeneAmp 9700, Applied Biosystems, Waltham, MA, USA) and amplicons were sequenced by an Illumina MiSeq platform at the Shanghai Majorbio Co. Ltd. (Shanghai, China) The raw sequencing data were deposited in the Sequence Read Archive database (https://www.ncbi.nlm.nih.gov/sra accessed on 20 April 2022) with the final SRA accession number PRJNA820348. The microbial diversity was further analyzed using the free online platform of the Majorbio Cloud Platform (https://www.majorbio.com accessed on 20 April 2022). In addition, the phylogenetic molecular ecological networks (MENs) were constructed to compare microbial interactions among different strategies.

## 3. Results and Discussion

### 3.1. Characterization and MFC Performance of S10-NFO@CC Nanosheets

**Figure 1:**
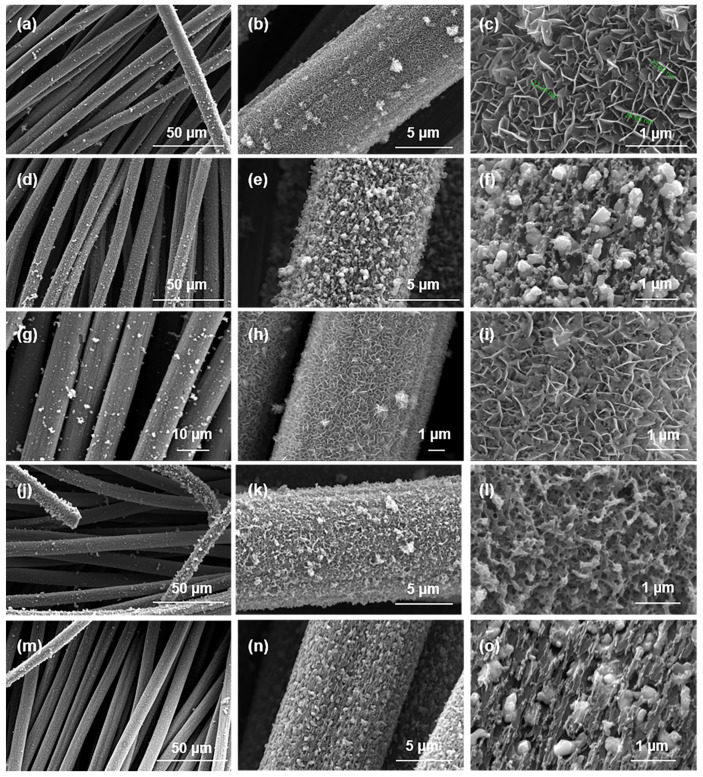
SEM images of (**a**–**c**) NFO400@CC, (**d**–**f**) NFO700@CC, (**g**–**i**) S10-NFO400@CC, (**j**–**l**) S10(T8)-NFO400@CC, (**m**–**o**) S10-NFO700@CC.

S-NiFe_2_O_4_ nanosheet arrays on carbon cloth (S-NFO@CC) was successfully synthesized by a simple hydrothermal sulfidation method. First, Fe^3+^ and Ni^2+,^ with the assistance of F^−^ as a structure-directing agent [31], reacted with CO_3_^2−^ and OH^−^, which were gradually released from urea during the hydrothermal reaction, led to the formation of NiFe-LDH nanosheets in situ arrays on carbon cloth (NiFe-LDH@CC). Subsequently, NiFe-LDH@CC was annealed to obtain NFO@CC. Then the sulfurization reaction within the presence of S^2−^ via anion-exchange process based on the Kirkendall effect [32,33,34] to achieve S^2−^ doping. The morphology of different NFO@CC materials is shown in Figure 1. Figure 1a–c show the whole carbon cloth substrate of NFO400@CC was covered closely and uniformly by nanosheets. Nevertheless, the hierarchical porous structure of nanosheets was destroyed under 700 °C, which can be significantly observed in NFO700@CC (Figure 1f). In addition, we can see S10-NFO@CC maintained the same morphology as the corresponding NFO@CC. However, when the sulfurization reaction time exceeded 8 h, the morphology of S10(T8)-NFO400@CC was destroyed (Figure 1l).

**Figure 2:**
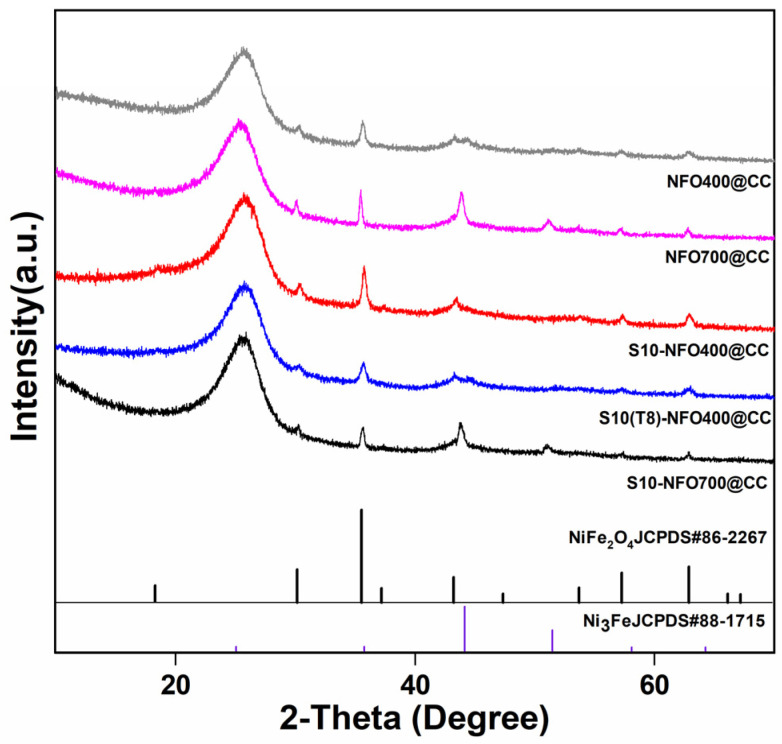
XRD patterns of NFO400@CC (gray curve), NFO700@CC (magenta curve), S10-NFO400@CC (red curve), S10(T8)-NFO400@CC (blue curve), S10-NFO700@CC (black curve).

The crystalline phases of NFO@CC and S10-NFO@CC nanomaterials were investigated by XRD. As shown in Figure 2, NFO400@CC displayed the same crystal structure as NiFe_2_O_4_ (JCPDS: 86-2267), and some extra diffraction peaks of NFO700@CC matched by FeNi_3_ (JCPDS: 65-5131), which shows the high-temperature calcination forms a new phase inside the NFO700@CC. Additionally, S10-NFO@CC displayed the same crystal structure as the corresponding NFO@CC, which implies the low-temperature sulfurization could not further introduce new phases inside the NFO@CC precursor [25].

**Figure 3:**
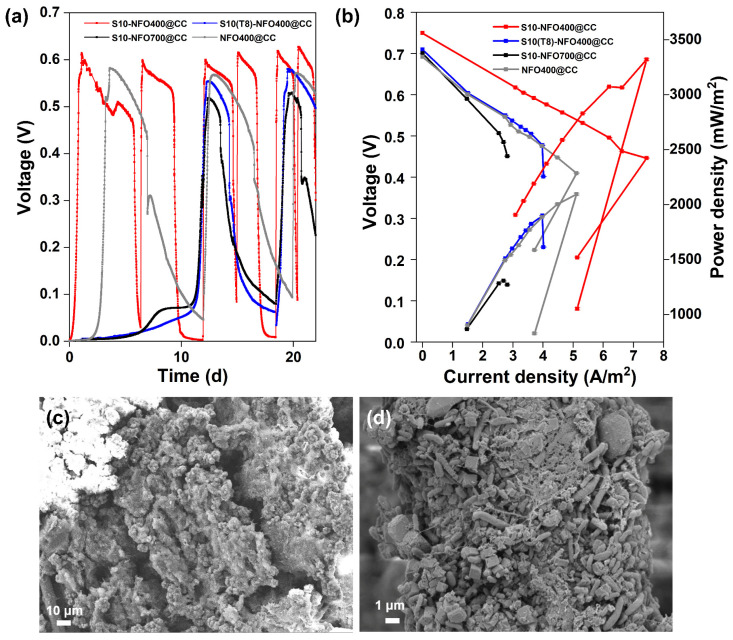
S10-NFO@CC as MFC anodes. (**a**) Start-up voltages, (**b**) Polarization and power density curves of S10-NFO400@CC (red curve), S10(T8)-NFO400@CC (blue curve), S10-NFO700@CC (black curve). SEM images of biofilm growth on (**c**) S10-NFO400@CC anode, and (**d**) S10(T8)-NFO400@CC anode.

Then, two-chamber MFCs were constructed to evaluate the electricity generation performance of different S10-NFO@CC anodes. Figure 3a displays the start-up voltages of S10-NFO@CC and NFO400@CC anodes. After inoculation for 3.6 d, a stable maximum voltage of 0.58 V was obtained for the NFO400@CC anode (gray curve). The start-up time was 1 d to achieve a maximum voltage of 0.61 V for the S10-NFO400@CC anode (red curve), while the start-up time was 12 d to obtain a maximum voltage of 0.55 V for S10(T8)-NFO400@CC anode (blue curve), and 12 d to obtain a maximum voltage of 0.52 V for S10-NFO700@CC (black curve), respectively. The time to obtain the maximum repeatable voltage of the S10-NFO400@CC anode, which is the shortest one in all S10-NFO@CC anodes, is 91.7% shorter than for two other S10-NFO@CC anodes. These show different sulfurization conditions affect MFC performance, and 3D porous nanosheet-modified surfaces are conducive to bacterial adhesion and faster construction of EET pathways.

After four cycles of the voltage generation, polarization and power density curves of anodes were tested and shown in Figure 3b. The open-circuit potential of the S10-NFO400@CC anode was the highest value (0.75 V), followed by S10(T8)-NFO400@CC anode (0.71 V), S10-NFO700@CC (0.70 V), and NFO400@CC anode (0.69 V). The maximum power density generated by the S10-NFO400@CC anode was 3320.78 mW m^−2^ with 300 Ω of external resistance (Figure 3b, red curve), 1.6 times more than with the NFO400@CC anode (2095.66 mW m^−2^, external resistance: 400 Ω, gray curve). On the contrary, the maximum power density generated by S10(T8)-NFO400@CC anode was 1901.05 mW m^−2^ with 600 Ω of external resistance (blue curve), a 9.3% decrease compared to the NFO400@CC anode. The maximum power density generated by the S10-NFO700@CC anode was 1306.74 mW m^−2^ with 900 Ω of external resistance (black curve), 0.62 times that of the NFO400@CC anode. Figure 3c,d show biofilms enriched on S10-NFO400@CC and S10(T8)-NFO400@CC, respectively. The morphology of the nanosheets was covered as the growth of the biofilm. Different bacteria wrapped around the anode and promoted EET, leading to stable electricity generation. To sum up, the S10-NFO400@CC surface has a higher EET ability, obviously improving the power generation of MFC.

### 3.2. Effects of S10-NFO400@CC Anode on Microbial Community in Suspensions

**Figure 4:**
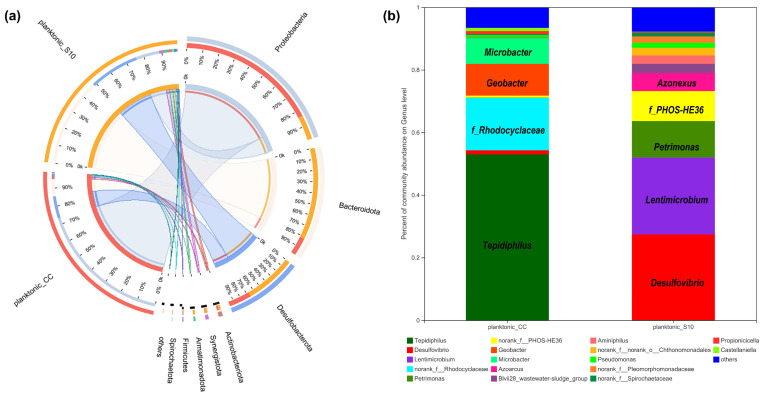
The relative abundance of microbial community compositions of planktonic_CC and planktonic_S10 at the phylum (**a**), and genus level (**b**), respectively.

We determined the microbial community of planktonic bacteria to analyze the effect of the S10-NFO400@CC anode on it. As shown in Figure 4a, at the phylum level, eight bacterial phyla were identified with over 1% relative abundance, and the dominating phyla were *Proteobacteria*, *Bacteroidetes*, *Desulfobacterota* in planktonic_CC and planktonic_S10, which were always found in the MFCs [35,36]. The difference is that *Proteobacteria* (75%) was the most predominant phyla in planktonic_CC, followed by *Desulfobacterota* (12%), *Bacteroidetes* (49%) were the uppermost in planktonic_S10, followed by *Desulfobacterota* (28%). Further identification at the genus level is illustrated in Figure 4b; *Tepidiphilus*, as the main component of *Proteobacteria*, were primarily enriched in the planktonic_CC (53.01%), followed by *f_Rhodocyclaceae* (16.74%), *Geobacter* (10.14%), and *Microbacter* (8.22%), whereas *Desulfovibrio* (27.36%), *Lentimicrobium* (24.59%), *Petrimonas* (11.64%), *f_PHOS-HE36* (9.57%), *Azonexus* (5.74%) in the anode solution gained favorable competition with S10-NFO400@CC anode. *Tepidiphilus*, belonging to the family *Hydrogenophilaceae,* was found as the dominant bacteria in extreme environments such as oil reservoirs [37,38]. The family of *Rhodocyclaceae* is also related to extracellular polymeric substances (EPS) production in aerobic granular sludge (AGS) systems [39]. *Geobacter* [40] and *Desulfovibrio* [41,42,43] were well-known EAB and widely found in MFCs. Additionally, *f__PHOS-HE36*, as sulfur-oxidizing bacteria (SOB), *Lentimicrobium*, as fermentative bacteria (FB), and *Azoarcus* are Gram-negative and strictly anaerobic bacteria, which have been detected as dominant microorganisms in the BESs [10,44,45]. Moreover, *Lentimicrobium* [46] and *Petrimonas* [47] are FB, which might synergetically interact with *Geobacter* to enhance hydrogen production.

**Figure 5:**
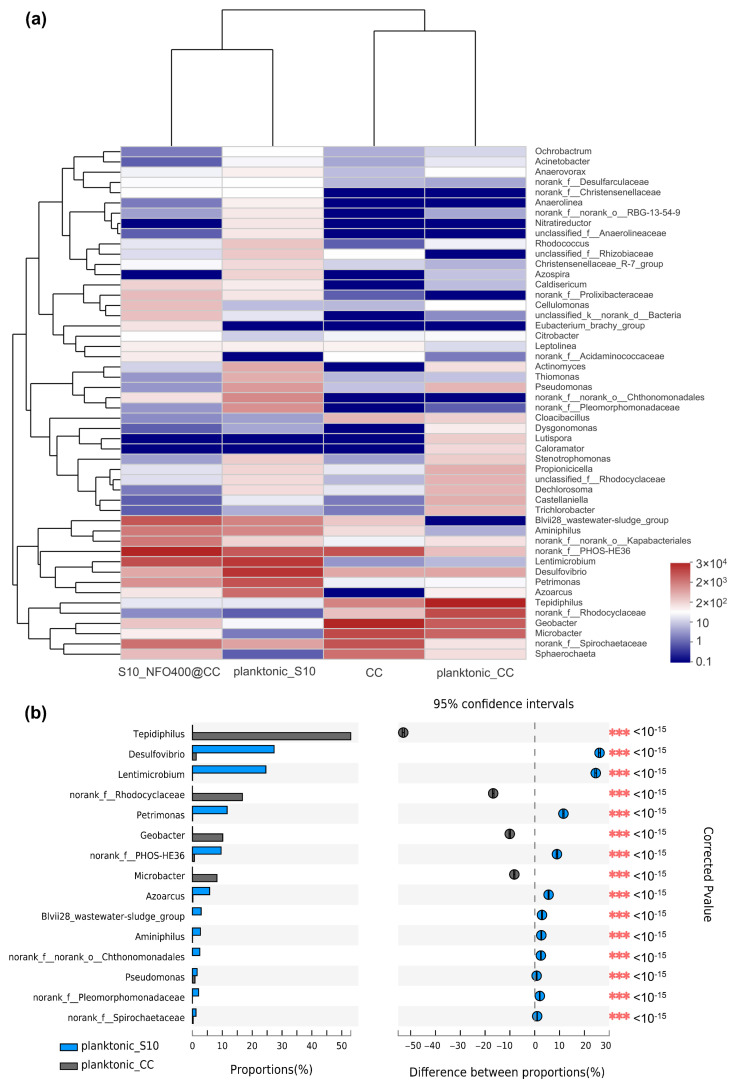
(**a**) The heatmap of the most abundant genera for anode biofilms and correspondingly planktonic bacteria. (**b**) Bacterial community of the planktonic bacteria had a significant difference (*** *p* ≤ 0.001) between CC anode and S10-NFO400@CC anode at the genus level.

As seen in the visual heatmap comparison (Figure 5a), the microbial community of anode biofilm was similar to that of suspension in the same MFC, while there were some variations in microbial community compositions at the genus level in response to different anode materials. Figure 5b specifically analyzes the differences in genus-level bacteria between those two planktonic bacteria. Compared to planktonic_CC, a significant increase in the relative abundance of 11 bacterial taxa and a significant decrease in 4 bacterial taxa were observed in planktonic_S10. Particularly, *Desulfovibrio*, *Lentimicrobium*, *Petrimonas*, and *f_PHOS-HE36* were significantly enriched in planktonic_S10. *Tepidiphilus*, *f_Rhodocyclaceae*, *Geobacter*, and *Microbacter* were suppressed.

### 3.3. Electricity Generation Performance of MFCs with Different Strategies

**Figure 6:**
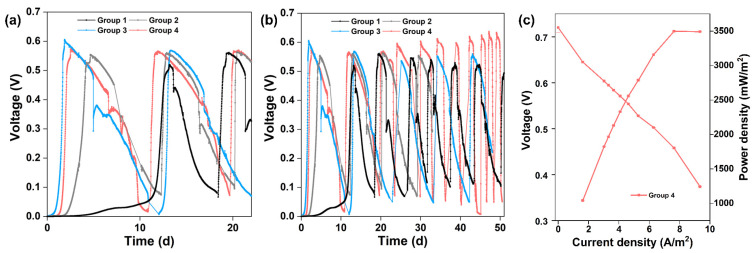
(**a**) Start-up voltages of different groups. (**b**) Long-term MFC stability of different groups. (**c**) Polarization and power density curves of Group 4.

New MFCs with CC anodes were operated by inoculating planktonic_CC or planktonic_S10. Figure 6a displays the start-up voltages of different strategies. The start-up time was 112 h to obtain a maximum voltage of 0.55 V for CC anode inoculated with planktonic_CC (Group 2, grey curve) and 45 h to achieve a maximum voltage of 0.60 V for CC anode inoculated with planktonic_S10 (Group 3, blue curve), respectively. The start-up time of Group 2 and Group 3 is shortened by 64.4% and 85.7% compared to that of CC anode inoculated with pre-acclimated bacteria (Group 1, black curve), respectively. It is suggested that planktonic_S10 contained more EAB with higher electrochemical activity, which could contribute to the rapid start-up of MFCs and achieve the same high voltage level of S10-NFO400@CC anode. After inoculation for 61 h, a maximum voltage of 0.57 V was obtained for Group 4 (pink curve). Group 4 and Group 3 were inoculated with the same planktonic_S10, but the start-up performance of Group 4 was worse than that of Group 3, which was directly related to the biofilm attached to the inner wall of the anode chamber (lining bacteria).

Long-term operation of MFCs was performed in different groups for two months. Figure 6b illustrates the consecutive electricity generation cycles of different groups. The maximum voltage of Group 2 was stable at 0.55 to 0.56 V, and surprisingly the maximum voltage of Group 4 exceeded 0.6 V after four cycles, up to 0.64 V after one month. Oppositely, the maximum voltage in Group 3 dropped continuously until it stabilized at 0.55 V after one month. The changes in maximum voltages reflect the competition between bacteria enriched on the anode surface and imply different outcomes of anode microbes. After four cycles of the voltage generation, the maximum power density generated by Group 4 was 3497.44 mW m^−2^ with 300 Ω of external resistance (Figure 6c), which is close to that of the S10-NFO400@CC anode (Figure 3b). In conclusion, the electricity generation performance of Group 4 with a common carbon cloth anode could achieve the same high level as the best S-doped nanomaterial (S10-NFO400@CC anode).

### 3.4. Microbial Community Analysis of Anode Biofilm with Different Strategies

**Figure 7:**
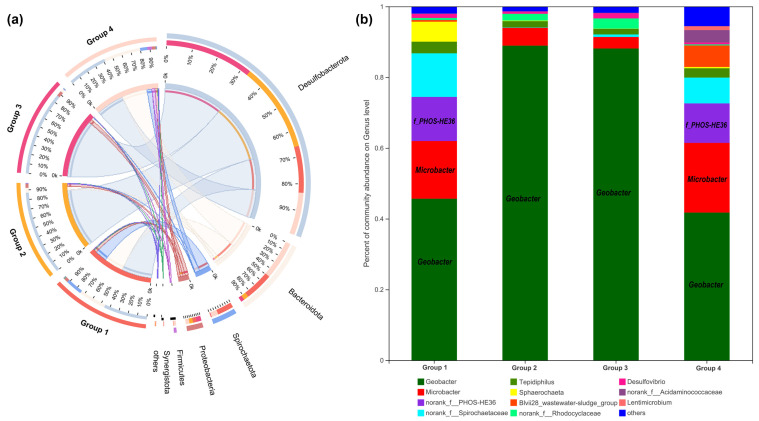
Microbial community analysis of anode biofilms in different groups. The relative abundance of microbial community compositions at the phylum (**a**), and genus level (**b**), respectively.

16S rRNA gene analysis was employed again to compare microbial communities residing in different anode biofilms. As shown in Figure 7a, at the phylum level, six bacterial phyla were identified with over 1% relative abundance and the dominating phyla were *Desulfobacterota*, *Bacteroidetes*, and *Spirochaetota*. Further identification at the genus level is illustrated in Figure 7b; *Geobacter* as the representative EAB was significantly enriched on anode biofilm of Group 2 (88.94%), followed by Group 3 (88.13%), Group 1 (45.67%), and Group 4 (41.75%). Another kind of EAB of *Microbacter* decreased in the anode biofilm of Group 2 (5.03%) and Group 3 (3.26%) and increased slightly in that of Group 4 (19.71%), compared to Group 1 (16.31%). Additionally, *f_PHOS-HE36* of anode biofilm was a similar amount in Group 1 (12.47%) and Group 4 (11.15%).

**Figure 8:**
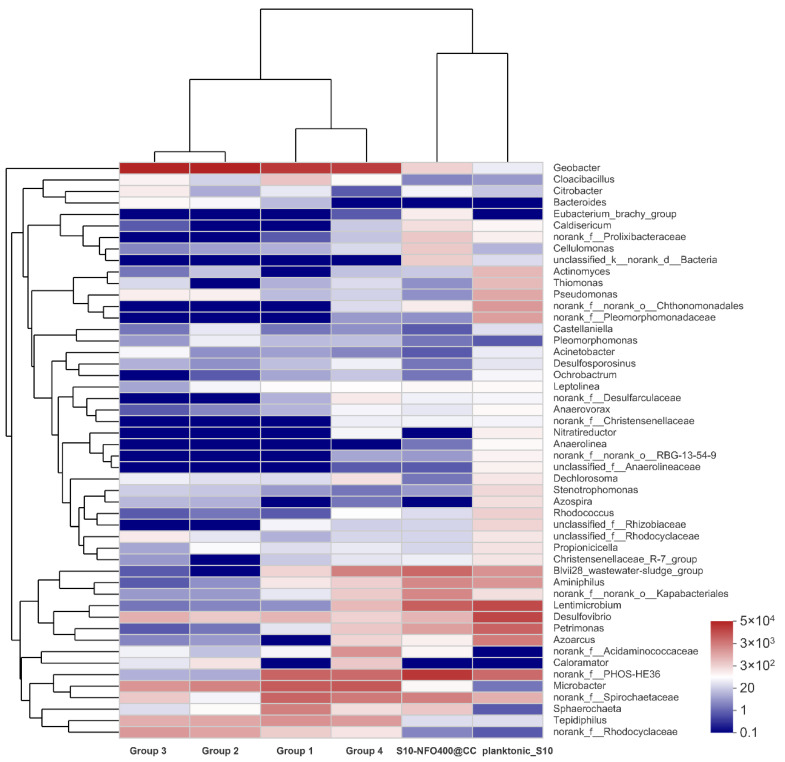
The heatmap of the most abundant genera for anode biofilm in different groups.

According to the visual heatmap further comparison shown in Figure 8, there were some variations in microbial community compositions at the genus level of different anode biofilms in response to different anode materials and the existence of microbes in different locations. Moreover, the community composition on the same anode material was also different. It was noticeable that the microbial community of the anode biofilm in Group 2 and Group 3 was similar with different inoculated bacteria and similar electricity generation performance. It hints that anode biofilms show a similar trend without other influence factors during MFC operation, which adheres and enriches more *Geobacter*, followed by *Microbacter* and *f_Rhodocyclaceae* under the stimulation of acetate. In addition, the microbial community of the anode biofilm in Group 1 and Group 4 was also similar with different inoculated bacteria and different electricity generation performance. It was illustrated that the existence of lining bacteria remembers the microbial composition of anode biofilm, and with the cooperation of lining bacteria and inoculated bacteria, a similar composition of microbes could be obtained. Moreover, the inoculated bacteria—planktonic_S10 could play a crucial role in the improvement of electricity production. However, both the microbial community of the anode biofilm and the electricity generation performance were different between Group 3 and Group 4 with the same inoculated bacteria. It also shows the existence of lining bacteria affects the development of anode biofilms. In brief, Group 4 remembers the microbial community composition of anode biofilms, owing to lining bacteria, and remembers electricity generation performance of S10-NFO400@CC anode, attributing to planktonic bacteria acclimated by S10-NFO400@CC anode, which can be called “the Memory Effect of MFCs”. Both lining bacteria and planktonic bacteria are indispensable for achieving “the Memory Effect of MFCs”.

### 3.5. Molecular Ecological Networks Analysis

**Figure 9:**
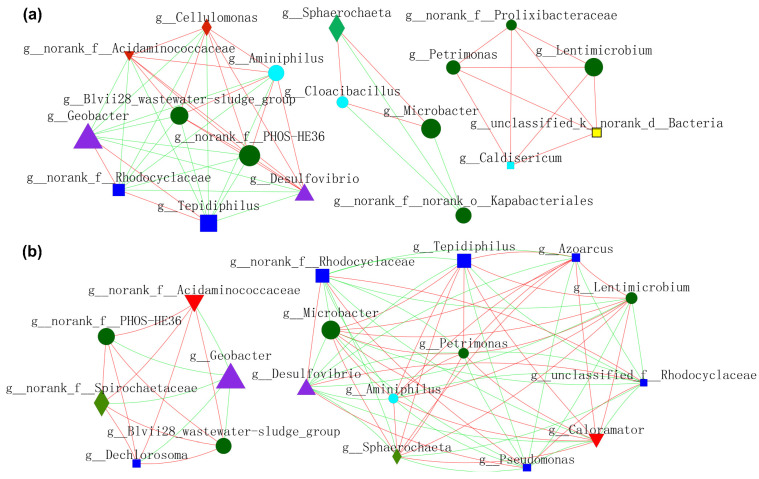
Molecular ecological networks (MEN) visualization of electroactive consortia in MFCs. (**a**) shows microbes resided in anode biofilm mediated by S-doped nanomaterials. (**b**) shows microbes resided in biofilm of CC anode in Group 2, Group 3 and Group 4. Different colors and shapes of nodes signifier categories of phylum: *Proteobacteria* (deep blue diamond), *Bacteroidota* (deep green round), *Desulfobacterota* (purple triangle), *Synergistota* (sky blue round), Unclassified phylum (yellow diamond, *Firmicutes* (red diamond), *Actinobacteriota* (red inverted triangle), *Spirochaetota* (light green diamond). A red edge indicates a positive interaction between two individual nodes, while a green edge indicates a negative interaction.

**Figure 10:**
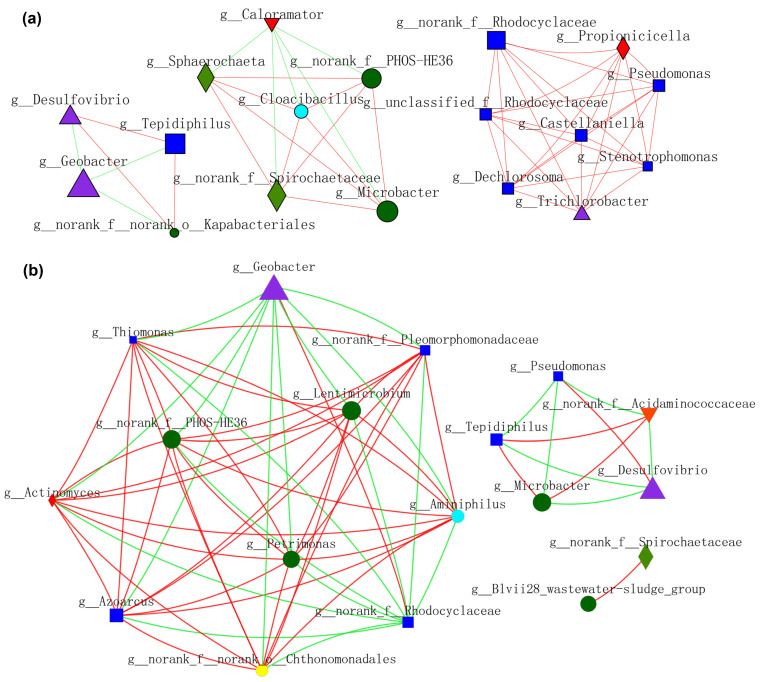
Molecular ecological networks (MEN) visualization dominated by (**a**) planktonic_CC and (**b**) planktonic_S10. Different colors and shapes of nodes signifier categories of phylum: *Proteobacteria* (deep blue diamond), *Bacteroidota* (deep green round), *Desulfobacterota* (purple triangle), *Synergistota* (sky blue round), *Armatimonadota* (yellow round), *Firmicutes* (red diamond), *Actinobacteriota* (red inverted triangle), *Spirochaetota* (light green diamond). A red edge indicates a positive interaction between two individual nodes, while a green edge indicates a negative interaction.

In order to gain a deeper insight into the nexus (competition or mutualism) of microbes during the operation of MFCs, molecular ecological networks (MENs) of microbial communities were specifically constructed (Figure 9 and Figure 10). EAB and FB appeared to have more diversified and complicated connectivity, indicating clear cooperation and competition. Figure 9a shows the relationship between microbes residing in anode biofilm mediated by NFO400@CC and S10-NFO400@CC anodes. *Geobacter* showed a more positive relationship with *Tepidiphilus* and *f_Rhodocyclaceae*, whereas the opposite relationships with others. Additionally, a positive correlation network was established between FB (*Lentimicrobium* and *Petrimonas*) and the other three bacteria. Figure 9b displays the nexus of microbes residing in the biofilm of CC anode after inoculating different suspensions (Group 2, Group 3, and Group 4). *Geobacter* showed a negative association with *f_PHOS_HE36* and other bacteria. FB (*Lentimicrobium* and *Petrimonas*), *Desulfovibrio*, and *Microbacter* appeared to have more complicated connectivity. *Tepidiphilus*, *Petrimonas*, *Azoarcus*, and *Microbacter* exhibited positive affiliations, whereas the negative relationships with *Desulfovibrio* and *f_Rhodocyclaceae*. Comparison between Figure 9a,b show S10-NFO400@CC promotes the cooperation between *f_PHOS_HE36* and *Desulfovibrio* and the cooperation between *Geobacter* and *f_Rhodocyclaceae*, respectively.

Microbial interactions constructed by planktonic_CC during MFCs operation are shown in Figure 10a. *Geobacter* showed a negative relationship with *Desulfovibrio*, *Tepidiphilus*, and *o_Kapabacteriales*, whereas *f_PHOS_HE36* exhibited a positive affiliation with *Microbacter* and others. Moreover, a positive correlation network was established between *f_Rhodocyclaceae*, *Pseudomonas*, and the other six bacteria. Microbial interactions constructed by planktonic_S10 during MFCs operation are shown in Figure 10b. *Geobacter* and *f_Rhodocyclaceae* exhibited a more positive association with each other, whereas a negative association with others. SOB (*f_PHOS-SE36*) showed high positive affiliations with FB (*Lentimicrobium* and *Petrimonas*) and *Azonexus*. *Microbacter* showed a negative relationship with *Desulfovibrio*. Comparing Figure 10a,b, we can see that planktonic_S10 acclimated by the S10-NFO400@CC anode could enhance the positive connections of FB and SOB, thus promoting IET inside electroactive consortia. It was noticeable that the cooperation between *Geobacter* and *f_Rhodocyclaceae* promoted by NMs is sustainable, according to Figure 9a and Figure 10b.

## 4. Conclusions

This study has demonstrated that the S10-NFO400@CC anode achieved high electricity generation of MFC, simultaneously affecting the microbial community structure in the solution. With the cooperation of lining biofilm and planktonic bacteria, the electricity generation performance of traditional CC anode could achieve the same high level of S10-NFO400@CC anode. Network analysis regulated by S-doped anode uncovered that the introduction of iron-based nanomaterials (NMs) made *Geobacter* positively interact with *f_Rhodocyclaceae*, improving the competitiveness of EAB consortium (*Geobacter* and *f_Rhodocyclaceae*). In addition, the assistance of lining biofilm enhanced the cooperation of SOB and fermenters, promoting interspecies electron transfer, thus constructing more efficient electroactive consortia. It also implies that although planktonic bacteria acclimated by NMs alone do not remain steady-state under electrical stimulation and assistance of acetate, microbes in a larger range may assist planktonic bacteria to dominate. More attention also should be paid to the wholeness and connection of microbes in the whole artificial system or natural environment, such as lining biofilm, which could not be ignored in the long-term bioelectrochemical system and has to be further identified. These findings provide a feasible strategy for low-risk and low-cost environmental applications of NMs.

## Figures and Tables

**Table 1:** The specific description of different strategies.

Group	Anode	Inoculated Bacteria	Reactors
1	Carbon cloth	Original bacteria	New MFC reactor
2	Carbon cloth	Planktonic bacteria acclimated by CC anode	New MFC reactor
3	Carbon cloth	Planktonic bacteria acclimated by S10-NFO400@CC anode	New MFC reactor
4	Carbon cloth	Planktonic bacteria acclimated by S10-NFO400@CC anode	Used reactor with CC anode, containing bacteria on the inner wall (lining bacteria)

## Data Availability

The datasets generated for this study can be found in Sequence Read Archive under BioProject, BioProject ID: PRJNA820348 (https://www.ncbi.nlm.nih.gov/ accessed on 20 April 2022).
